# Detection of Viral Pathogens With Multiplex Nanopore MinION Sequencing: Be Careful With Cross-Talk

**DOI:** 10.3389/fmicb.2018.02225

**Published:** 2018-09-19

**Authors:** Yifei Xu, Kuiama Lewandowski, Sheila Lumley, Steven Pullan, Richard Vipond, Miles Carroll, Dona Foster, Philippa C. Matthews, Timothy Peto, Derrick Crook

**Affiliations:** ^1^Nuffield Department of Medicine, University of Oxford, Oxford, United Kingdom; ^2^National Institute for Health Research Oxford Biomedical Research Centre, University of Oxford, Oxford, United Kingdom; ^3^National Infection Service, Public Health England, Salisbury, United Kingdom; ^4^Nuffield Department of Medicine, Peter Medawar Building for Pathogen Research, University of Oxford, Oxford, United Kingdom; ^5^Department of Infectious Diseases and Microbiology, Oxford University Hospitals NHS Foundation Trust, John Radcliffe Hospital, Oxford, United Kingdom

**Keywords:** nanopore sequencing, metagenomics, multiplexing, cross barcode contamination, chimera

## Abstract

Metagenomic sequencing with the Oxford Nanopore MinION sequencer offers potential for point-of-care testing of infectious diseases in clinical settings. To improve cost-effectiveness, multiplexing of several, barcoded samples upon a single flow cell will be required during sequencing. We generated a unique sequencing dataset to assess the extent and source of cross barcode contamination caused by multiplex MinION sequencing. Sequencing libraries for three different viruses, including influenza A, dengue, and chikungunya, were prepared separately and sequenced on individual flow cells. We also pooled the respective libraries and performed multiplex sequencing. We identified 0.056% of total reads in the multiplex sequencing data that were assigned to incorrect barcodes. Chimeric reads were the predominant source of this error. Our findings highlight the need for careful filtering of multiplex sequencing data before downstream analysis, and the trade-off between sensitivity and specificity that applies to the barcode demultiplexing methods.

## Introduction

Metagenomic sequencing has the potential to allow unbiased identification of pathogens from a clinical sample. It holds the promise to serve as a single and universal assay for diagnostics of infectious diseases directly from samples without the need for *a priori* knowledge (Bibby, 2013; Miller et al., 2013; Schlaberg et al., 2017). In addition to identification of pathogen species, broad and deep metagenomic sequence data could provide information relevant to determining treatment and prognosis, detecting outbreaks and tracking infection epidemiology (Greninger et al., 2010; Yang et al., 2011; Qin et al., 2012; Loman et al., 2013). Next-generation sequencing (NGS) platforms can produce a massive throughput of data at a modest cost, however, its application in clinical diagnostic and public health has been limited by complexity, slowness, and capital investment.

The MinION is a palm-size, real-time, single-molecule genome sequencer developed by Oxford Nanopore Technologies (ONT). The MinION’s compact size and real-time nature could facilitate the application of metagenomic sequencing in point-of-care testing for infectious diseases, as demonstrated by several proof-of-concept studies, including identification of Chikungunya (CHIKV), Ebola (EBOV), and hepatitis C virus (HCV) from human clinical blood samples without target enrichment (Greninger et al., 2015), and detection of bacterial pathogens from urine samples (Schmidt et al., 2016) and respiratory samples, without the need for prior culture (Pendleton et al., 2017).

The data throughput of MinION has greatly increased since its release in 2015, with each consumable flow cell now generating up to 10–20 Gb of DNA sequence data. This allows users to make more efficient use of the flow cell (and reduce cost) by multiplexing several samples in a single sequencing run. ONT has developed PCR-free barcode sets that allow multiplexing of up to 12 samples.

Detection of influenza A virus in multiple respiratory samples could be one diagnostic use of a multiplexed MinION sequencing assay. However, when sequencing directly from samples with a potential wide range of viral titres, it is important to be aware of the potential for cross sample contamination, both during library preparation and the bioinformatic barcode demultiplexing stage following sequencing. Here, we present a unique MinION sequencing dataset and results of investigation into the extent and source of cross barcode contamination in multiplex sequencing.

## Materials and Methods

We used a ferret nasal wash sample infected with influenza A virus as an exemplar and also spiked two aliquots of negative nasal wash samples from uninfected ferret (pre-existing unused stocks from an unrelated study) with dengue and chikungunya viruses separately. Neither of these viruses are relevant for clinical diagnostics in respiratory samples, but act here as clear, distinct markers for the assessment of cross sample contamination. The sequencing libraries for each sample were prepared in parallel, along with a negative nasal wash control, barcoded, and sequenced individually. We then pooled an aliquot of the sequencing libraries and performed multiplex MinION sequencing. Reads from the four individual runs (referred to as “CHIKV,” “DENV,” “FLU-A,” and “Negative”) and the multiplex run (referred to as “Multiplexed”) were then analyzed to investigate the extent and source of cross sample contamination.

### Sample Preparation

The project license was reviewed by the local AWERB (Animal Welfare and Ethics Review Board) and was subsequently granted by the Home Office. RNA was extracted, using the QIAamp viral RNA kit (Qiagen) according to the manufacturer’s instructions, from ferret nasal wash containing influenza A (H1N1) virus (A/California/04/2009) and a pool of negative nasal wash samples. Aliquots of negative sample extract were spiked with either dengue (DENV) (strain TC861HA, GenBank: MF576311) or CHIKV (strain S27, GenBank: MF580946.1) viral RNA from The National collection of Pathogenic Viruses ^1^. Samples were DNase treated using TURBO DNase (Thermo Fisher Scientific, Waltham, MA, United States) and purified using the RNA Clean & Concentrator^TM^-5 kit (Zymo Research). cDNA was prepared and amplified using a Sequence-Independent-Single-Primer-Amplification methods (Greninger et al., 2015) modified as described previously (Atkinson et al., 2016). Amplified cDNA was quantified using the Qubit dsDNA HS Assay Kit (Thermo Fisher Scientific, Waltham, MA, United States), and 1 μg was used as input for each MinION library preparation, with the exception of the negative control where the entire sample (32 ng) was used.

### MinION Library Preparation and Sequencing

**FIGURE 1:**
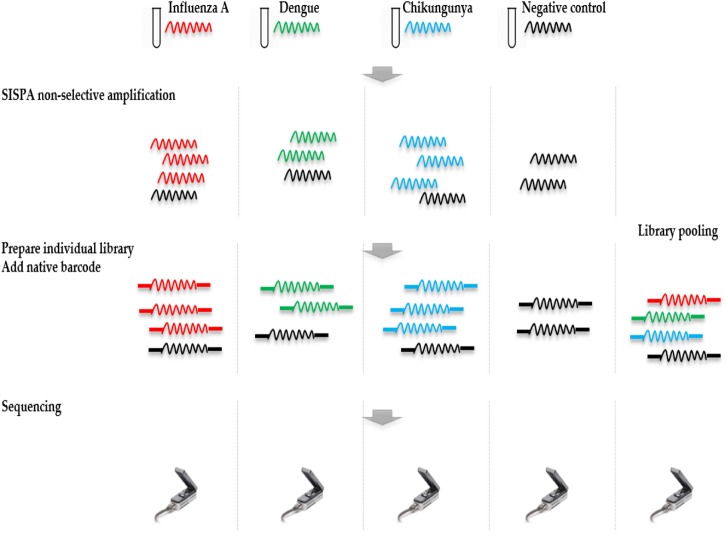
Overview of study design. RNA was extracted from four samples, including a ferret nasal wash sample infected with influenza A virus, two negative ferret nasal wash samples spiked with dengue and chikungunya viruses, and a negative ferret nasal wash control. cDNA was prepared and amplified using a Sequence-Independent-Single-Primer-Amplification methods. The sequencing libraries for each sample were prepared in parallel, barcoded, and sequenced on individual flow cells. Multiplex sequencing was also performed by pooling the four individual libraries. Reads from the four individual runs and the multiplex run were analyzed to assess the extent and source of cross barcode contamination in multiplex sequencing.

Ligation Sequencing Kit 1D (SQK-LSK108) and Native Barcoding Kit 1D (EXP-NBD103) were used according to the ONT standard protocols, with the exception that only one barcode was included in each of the four library preparations. Each library was run on an individual flow cell and a fifth pooled library was made by combining the four individually barcoded libraries. Libraries were sequenced on R9.4 flow cells. The study design is shown in **Figure 1**.

### Genomics Analysis

Reads were basecalled using Albacore v2.1.7 (ONT) with barcode demultiplexing. Reads from each sequencing run were mapped to genomic sequences of each virus using Minimap2 (Li, 2018). The number of reads mapped to reference was counted using Pysam^2^. *De novo* assembly was performed using Canu v1.7 (Koren et al., 2017), and the resulting draft genome was polished using Nanopolish (Mongan et al., 2015) with the signal-level data.

To allow stringent barcode demultiplexing of the multiplex MinION sequencing data, we performed two rounds of analyses using Porechop (v0.2.2^3^). Presence of adapter sequence in the middle of a read is a signature of chimera. We used Porechop to examine each read and those have middle region sharing >75% identity with adapter sequence were identified as chimeric reads. In Porechop, we set the “–middle_threshold” option and choose a threshold of 75. In the second round, we used Porechop to look up barcode sequence at both the start and end of a read; reads were assigned only if same barcode was found at two ends. We set the “–require_two_barcodes” option in Porechop and set the threshold for barcode score as 70. To find potential signature of chimeric reads, we examined the read current signals stored in the FAST5 file by MinION sequencer. Current signals were extracted using ONT fast5 API^4^ and plotted by using ggplot2 implemented in R^5^ for a comparison of chimeric and non-chimeric reads.

## Results

### MinION Sequencing Data and Assembly of Viral Genomes

The throughput of each MinION sequencing run varied due to differences in running time. A maximum number of ∼2.4 M reads was achieved by the multiplexed sequencing run and the individual CHIKV run, due to longer running times (**Supplementary Table S1**). Reads from the spiked virus accounted for 96% of the data in the individual CHIKV and DENV sequencing runs, and 78% for the FLU-A sample (**Table 1**). The percentage of viral reads within each barcoded sample in the Multiplexed sequencing data is close to that in the individually run sample data (**Table 2**). Each viral genome had an ultra-high (>8,000) mean depth of coverage in the individual and multiplex sequencing data, and *de novo* assembly was able to recover nearly complete genomes for all three viruses with 99.9% identities compared to the GenBank reference.

**Table 1:** Summary of mapping and *de novo* assembly results for data from MinION sequencing of individual libraries.

		Mapping			*De novo* assembly	
	Segment	Reads mapped (%)	Genome coverage (%)	Mean depth of coverage	Genome coverage (%)	Mismatch/Gap/Genome length
Chikungunya		96	100	160,000	99	2/4/11774
Dengue		96	100	75,000	99	4/6/10709
Influenza A	PB2	18	100	125,000	100	0/0/2280
	PB1	12	100	87,000	95	0/1/2274
	PA	11	100	84,000	100	0/0/2151
	HA	16	100	140,000	100	1/6/1701
	NP	11	100	114,000	99	0/2/1497
	NA	5	100	51,000	100	0/1/1410
	M	1	100	15,000	100	0/1/972
	NS	1	100	17,000	100	0/1/838

*The eight gene segments of influenza A virus are PB2, polymerase basic 2; PB1, polymerase basic 1; PA, polymerase acidic; HA, hemagglutinin; NP, nucleoprotein; NA, neuraminidase; M, matrix; NS, non-structural.*

**Table 2:** Summary of mapping and *de novo* assembly results for data from multiplex MinION sequencing.

		Mapping			*De novo* assembly	
	Segment	Reads mapped (%)	Genome coverage	Mean depth of coverage	Genome coverage (%)	Mismatch/Gap/Genome length
Chikungunya		94	100	42,000	99	1/21/11774
Dengue		95	100	51,000	99	2/8/10709
Influenza A	PB2	18	100	61,000	97	2/4/2280
	PB1	12	100	43,000	100	0/3/2274
	PA	11	100	41,000	100	0/0/2151
	HA	15	100	68,000	100	1/2/1701
	NP	10	100	55,000	100	2/4/1497
	NA	5	100	26,000	100	0/1/1410
	M	1	100	8,000	99	0/0/972
	NS	1	100	9,000	100	0/1/838

*The eight gene segments of influenza A virus are PB2, polymerase basic 2; PB1, polymerase basic 1; PA, polymerase acidic; HA, hemagglutinin; NP, nucleoprotein; NA, neuraminidase; M, matrix; NS, non-structural.*

### Extent and Source of Cross-Sample Contamination

**FIGURE 2:**
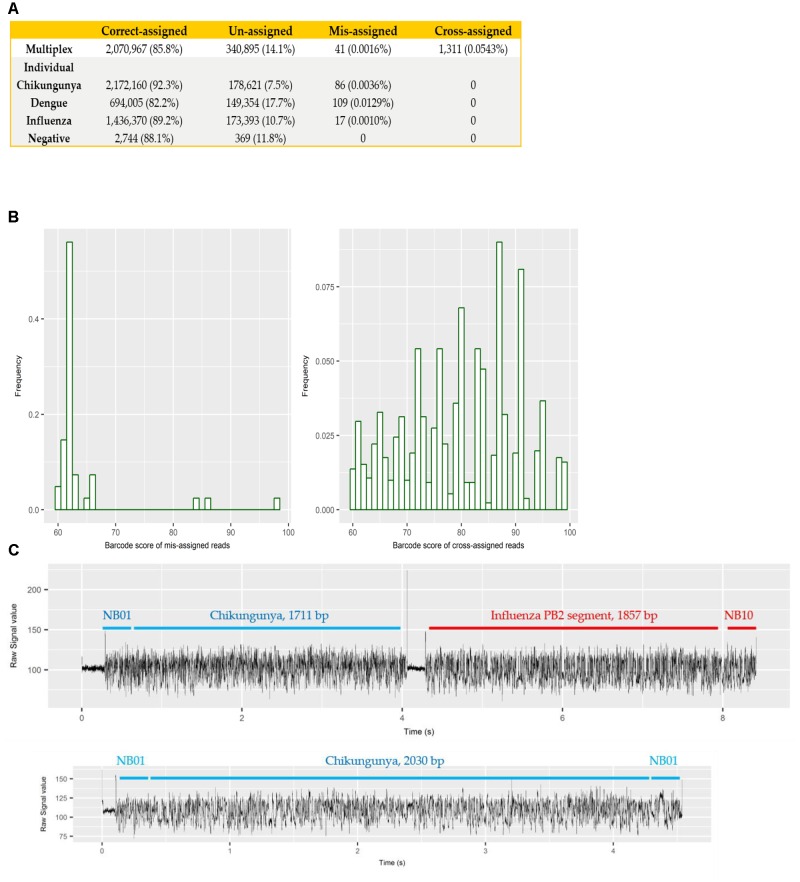
**(A)** summary of number and percentage of reads correctly assigned, unassigned, mis-assigned, and cross-assigned in each sequencing run. Un-assigned refers to reads that cannot be assigned to any bins by Albacore due to a barcode score less than 60, mis-assigned refers to reads that were assigned to barcode bins not included in this experiment, and cross-assigned refers to reads that were assigned to the incorrect barcode bins; **(B)** distribution of barcode scores reported by Albacore for mis-assigned reads and cross-assigned reads in the multiplex sequencing data; **(C)** comparison of raw signal of a chimeric and a correctly assigned read. The signal of chimeric read possesses a stall signal and a huge spike signal in the middle of the read.

Each sample was barcoded, and sequenced both individually and multiplexed, which allowed us to examine the performance of barcode demultiplexing of Albacore. In the individually sequenced sample data we would expect only a single native barcode to be present. For CHIKV (barcode NB01), DENV (NB09), and FLU-A (NB10) individual sequencing runs, we found that 86, 109, and 17 reads, respectively, were assigned to barcode bins not expected to be present in the library (representing 0.0036, 0.0129, and 0.001% of total reads). In the multiplex sequencing data, 41 reads (0.0016%) were assigned to barcodes not included in the experiments (i.e., a barcode other than NB01, NB05, NB09, or NB10). We defined these as mis-assigned reads (**Figure 2A**).

To examine potential laboratory contamination in sequencing library preparation, we mapped all reads from each individual run against the genomic sequences of all three viruses. No read was found to originate from a genome prepared in a different library, suggesting no *in vitro* contamination. The multiplex sequencing library was prepared by pooling the individual, non-contaminated libraries after the ligation of both barcode and adapter. However, mapping results show 1,311 (0.0543%) reads mapped to the incorrect target genome, implying that they were cross-assigned to the wrong barcode bins (later referred to as “cross-assigned reads”), despite the fact that the multiplexed sequencing library was pooled with individual libraries showed no cross-assigned reads at all. We hypothesized that mis-assigned and cross-assigned reads were due to a low barcode score, and investigated the barcode scores of these reads. Most of the mis-assigned reads had a barcode score <70, however, cross-assigned reads had more diverse scores ranging from 60 to nearly 100 (**Figure 2B**). This result suggested that mis-assigned and cross-assigned reads originate from different sources. We blasted the cross-assigned reads to a small database comprising the genomic sequences of the three viruses included in this study, and demonstrated that 1074/1311 (82%) of these reads could be cross aligned to more than one viral genome (1,047 reads) or cross aligned to distinct regions within the same genome (27 reads), suggesting they are chimeras. To confirm this observation, we investigated the raw current signals of a few cross-assigned reads compared to those of correctly assigned reads (**Figure 2C**). The current signals of a correctly assigned read usually include: (i) an open pore signal of high current representing the time that the sequencing pore changes from one adapter to another, (ii) a stall signal, referring to the period of time that a DNA sequence is in the pore but yet to move, and (iii) the signal trace of DNA sequencing. In contrast, a chimeric read possesses a stall signal and a huge spike signal in the middle of the read. Chimeric reads can possess two different barcode sequences at the start and end, thus confusing assignment of a barcode bin. Taken together, these data demonstrate two categories of error that contribute to cross sample contamination in our dataset: (i) chimeric reads (account for ∼80% of all cross-assigned reads); (ii) reads with low barcode score. In order to improve the quality of our final dataset, we explored the impact of different barcode demultiplexing approaches to remove cross-assigned reads (**Table 3**). Filtering of the reads that possess an internal adapter can remove 90% of the cross-assigned reads and lost 24% of total reads. We also tried a more stringent filtering scheme that required two barcodes (one each at the start and end of the read) to make an assignment. This approach removed all but two cross-assigned reads, but lost 56% of total reads.

**Table 3:** Removal of cross-assigned reads and loss of total sequencing data by two filtering approaches using Porechop.

	Total reads		Cross-assigned reads	
	Unclassified	Classified	Removed	Retained
Before filtering	340,895 (14%)	2,072,278 (86%)	0 (0%)	1,311 (100%)
Filter chimeric reads	591,131 (24%)	1,822,042 (76%)	1,176 (90%)	135 (10%)
Require two barcodes	1,351,028 (56%)	1,062,145 (44%)	1,309 (99.8%)	2 (0.2%)

*The first approach examines internal adapter and filter chimera candidates that possess adapter in the middle of the read. The second approach requires two barcodes at the start and end of each read. Increasing stringency for barcode demultiplexing occurs at the expense of reducing total number of reads.*

We also investigate the extent of potential chimeric reads in the sequencing data. For CHIKV, DENV, and FLU-A individual sequencing runs, mapping results show that 2.3, 3.0, and 2.7% of mapped reads, respectively, possess supplementary alignment and aligned at least twice to the same genome (**Table 4**). We consider both the barcode classified and unclassified reads in the multiplex sequencing data. Results show that 2.0% of mapped reads possess supplementary alignment and aligned at least twice to the same genome, while 0.052% of total reads were aligned to at least two distinct genomes.

**Table 4:** Summary of number and percentage of non-chimeric, self-chimeric, and cross-chimeric reads in each sequencing run.

	Non-chimeric	Self-chimeric	Cross-chimeric
Multiplex	2,048,917 (98.0%)	42,811 (2.0%)	1,097 (0.052%)
Individual			
Chikungunya	2,226,369 (97.7%)	53,316 (2.3%)	0
Dengue	789,009 (97.0%)	24,012 (3.0%)	0
Influenza	1,229,770 (97.3%)	33,858 (2.7%)	0
Negative	0	0	0

*Self-chimeric refers to reads that possess supplementary alignment and aligned at least twice to the same genome; cross-chimeric refers to reads that were aligned to at least two distinct genomes.*

## Discussion

The ultimate objective of our research is to develop a nanopore metagenomic sequencing based diagnostic assay that enables point-of-care testing for infectious diseases. Multiplex sequencing offers the opportunity to improve scalability and cut cost, however, cross sample contamination can lead to errors in the data and false interpretation of the results.

In this experiment, we pooled clean libraries and performed multiplex MinION sequencing in order to investigate the extent and source of cross-barcode contamination. We identified 0.056% of total reads were cross-assigned to the incorrect barcode bins, which is comparable to those reported for Illumina sequencing platforms from different studies (between 0.06 and 0.25%) (Nelson et al., 2014; D’Amore et al., 2016; Wright and Vetsigian, 2016). Our results showed that chimeric reads are the predominant source of cross-barcode assignment errors. Cross-assigned chimeric reads in this dataset could only have been formed during sequencing rather than library preparation, as they were completely absent in the sequencing data of individual libraries, and the only further processing step was to mix the final sequencing libraries prior to loading. We hypothesize that the current algorithm implemented in Albacore cannot recognize the short dissociation between DNA sequences that run concurrently through the nanopore, thereby concatenating more than one sequence into the same Fast5 file.

Chimeric reads were observed in MinION sequencing data before in White et al. (2017). Through analyses of the MinION sequencing data of three different interferon amplicons, the authors found that 1.7% of mapped reads were chimera. Our findings add to the knowledge supporting that chimera are common in MinION sequencing data. We identified between 2 and 3% of total reads in three individual and one multiplex sequencing data are chimera. Our study differs from previous work in the following two aspects. First, we provide direct evidence that chimeric reads can be formed after library preparation and during sequencing; we further linked these chimera to cross-sample contamination in multiplex MinION sequencing as discussed above. On the other hand, our experiment setup has limitation in identify potential chimera formed in library preparation, particular during the adaptor ligation step in the standard multiplex sequencing protocol. Second, our findings reflect the current status of MinION sequencing because we used newer and most representative ONT sequencing kit, including ligation sequencing kit 1D (SQK-LSK108) and native barcoding kit 1D (EXP-93 NBD103). Nanopore sequencing technology is under rapid development and improvement is happening in all aspects. For example, newer DNA ligation sequencing kit (SQK-LSK109) and direct RNA sequencing kit (SQK-RNA001) have been released; basecalling algorithm implemented in Albacore and Guppy basecaller has been upgraded. All these changes have effect on the extent of chimera in Nanopore sequencing data and cross-barcode contamination during multiplex sequencing. The limitation of this study was the small number of experiment, additional work using different experiment setups would add to our understanding of Nanopore multiplex sequencing data. In addition, it is important to investigate the contributions of potential factors to cross-barcode contamination, which would shed light on best practice to analyze multiplex sequencing data.

In summary, our study demonstrated that chimeric reads are the predominant source of cross barcode assignment errors in multiplex MinION sequencing. It highlights the need for careful filtering of multiplex MinION sequencing data before downstream analysis, and the trade-off between sensitivity and specificity that applies to the barcode demultiplexing methods.

## Author Contributions

SP, KL, SL, and YX conducted MinION sequencing. YX analyzed the data. All authors designed the study, participated in interpreting the results and writing the manuscript, and read and approved the final version of this manuscript.

## Conflict of Interest Statement

The authors declare that the research was conducted in the absence of any commercial or financial relationships that could be construed as a potential conflict of interest.

## Footnotes

1 www.phe-culturecollections.org.uk

2 https://github.com/pysam-developers/pysam

3 https://github.com/rrwick/Porechop

4 https://github.com/nanoporetech/ont_fast5_api

5 https://www.r-project.org/
