# Supplementary material for: Detection of Viral Pathogens With Multiplex Nanopore MinION Sequencing: Be Careful With Cross-Talk
Source: Front Microbiol. 2018 Sep 19;9:2225. doi: 10.3389/fmicb.2018.02225 (PMC6156371; doi:10.3389/fmicb.2018.02225)
Supplement: Supplementary file 1 [file Table_1.docx]

**Supplementary table**

**Table S1** Summary of statistics of MinION sequencing data.

|  | **Virus** | **Barcode** | **SISPA concentration (ng/µl)** | **Number of reads** | **Total length (bp)** |
| --- | --- | --- | --- | --- | --- |
| **Individual sequencing** | chikungunya | NB01 | 88.6 | 2,350,867 | 2,319,958,089 |
|  | dengue | NB09 | 70.2 | 843,468 | 965,165,841 |
|  | influenza A virus | NB10 | 49.4 | 1,609,780 | 1,749,616,221 |
|  | negative control | NB05 | 0.712 | 3,113 | 3,475,147 |
| **Mutliplex sequencing** | chikungunya | NB01 | - | 639,986 | 632,952,453 |
|  | dengue | NB09 | - | 580,183 | 666,239,544 |
|  | influenza A virus | NB10 | - | 823,427 | 881,448,974 |
|  | negative control | NB05 | - | 28,682 | 29,967,359 |
|  | unclassified |  |  | 340,895 | 360,996,367 |
